# Always against the Woman

**Published:** 1879-04

**Authors:** Ausburn Towner


### ALWAYS AGAINST THE WOMAN.

**The Second of Two Wrongs That Sadly Needs Righting.**

BY AUSBURN TOWNER.

PART II.

A discussion concerning the second wrong of which I would speak, that deserves redress, has an element of the ludicrous in it, as it is almost impossible to refer to clothes in a serious mood, unless you are a mantua-maker or tailor, a fop or an ultra fashionable woman. That is clothes in the abstract. Clothes in the concrete are very serious considerations for many people, especially impecunious mothers and fathers of large families. The wrong that appertains to them is that legally you are not allowed, even in this land of Christian enlightenment, to dress as you please, and that it falls hard upon women that it is so, without materially affecting men. At the broad statement, one who had not examined the question, would be excusable for rubbing his eyes and wondering what is meant. He would naturally, if much of a reader, call to mind the old days when the shape, color, length and cut of a garment were determined by law; when the Parliaments of Edward III., Henry VII. and Henry VIII., prescribed how many yards a woman might have in her petticoat, how deep her ruffles, how high her head-dress; when they forbade the men to wear shoes so long in the toes that they could be twisted around the waist, or passed those, to us, supremely ridiculous enactments condemning a man to the pillory for wearing a shirt. And contrasting these with the present day, which shows such an immense variety in garments, with no one to criticize or condemn save rivals or inferiors, you would be excusable for denying that there was any force in the averment that you are not allowed to dress as you choose. Yet there is still a limit put to your tastes or desires, that is made just as binding and is quite as ridiculous as any of the ancient enactments, of which it is really about the last remaining remnant. You man, try to appear in public, it would not perhaps be commendable in you to desire to do so, although there is nothing inherently wrong in it, in the garments of your sister or wife, or you woman, dress up in your husband's or brother's clothes, and see how many blocks you would be able to go without being dragged before some magistrate and compelled to pay a fine or go to jail. Does it not therefore hold good that you are not allowed to dress as it pleases you?

I have sought diligently for the reasons that underlie the law, and for arguments to sustain it, but I can find none. It is said to be a Bible command that men should not wear women's garments, nor women men's. But if so, the direction was given in times entirely different from these and no more applies now-a-days than do the old laws and manual of arms that governed the handling of bows and arrows, pikes and halberds. The saying that is as old as the time of Joan of Arc, and older, that she unsexed herself by putting on men's garments is simply ridiculous. Clothes by no means make or mark in reality the difference in sex. Call up the flowing togas of the old Romans or the broad skirts of the Jewish Priests and see what they say and interrogate the trousers that eastern women wear, for an answer.

The reason of the statutory provision, if there is one, is that persons thus disguised might do some immoral or criminal act, and their being so disguised is taken as presumptive evidence that such an act is contemplated. Even the law is not willing to take the position that any dress any one may choose to put on is wrong in itself, but hides behind a supposition. Why don't the Legislature, on the same grounds, stop the building of the East River Bridge? It will be a temptation to some to jump from it and commit suicide, and doubtless many will do so. Or why is not the principle carried out in every direction? Here goes along a person with a leathern bag in his hand. The bag flies open and there drops out a full set of burglar's tools. Now no police officer would have the right to arrest that man, yet the supposition would be much stronger of a crime contemplated than if, instead of his tools, he had had on a woman's dress. Yet in the latter case, the officer could have taken him in.

I would be obliged to any one who would point out to me any logical reason, physiological, moral or natural, why the garments of one sex are not suitable for the other.

On the other hand, I think it easy to be demonstrated, that the law or regulation prohibiting women from wearing men's clothes, is unreasonable and markedly unjust.

I do not contend that it is commendable for a man to wear, or wish to wear petticoats, or for a woman in all cases to wear trousers, but it is for their right to do so if they choose. With the first, in fact, I have no sympathy whatever, but with the latter, inasmuch as the prohibition strikes both ways, a great wrong is worked that manifests itself every day.

The proposition that every one should be allowed to dress as he pleases, so long as he does not outrage decency, or interfere with the rights of others and pays for what he wears, is one that no one can dispute. It is in harmony with the largest liberty, and so in harmony with all our institutions. Dress is something that regulates itself, and laws have no more business with it than they have with what a man should eat or drink, or in what shape or style he shall build his house. It is like trade or money, entirely above and beyond the control of Legislative and statutory enactments.

A point might be touched on here and, perhaps, deserves to be alluded to, although it may be trite, the suggestion of indelicacy that bifurcated garments upon a woman puts forth. It only needs to be stated to see its falsity. A man's dress upon a woman is anything but indelicate. A man's dress is, in itself, ugly. It conceals the figure entirely; it covers up every line of beauty in the form and squares every curve to rectangular preciseness. From the hard hat to the harder boots a man's dress is indicative of the prim, puritanical notions in which it had its origin, and the finest formed woman in the world, even Phryne herself could not add grace to it or invest it with anything else.[1]

And therein lies one of the most unreasonable points of the law. It is woman's nature to desire to be attractive to men, to obtain their admiration, and this comes first through the eye. They would never put on a man's dress to do this. Where would be the opportunity for the display of a round arm or a white and plump neck? No. They dislike a man's dress and unless in cases like Dr. Mary Walker or Mrs. Tom-n-John, who are a kind of nondescripts, they never adopt it as a permanent apparel. Are there mountains to be scaled, [2] or is there a sea-bath to be taken, or are they seeking other things which are yet to be referred to, they put on a man's dress, or a near approach to it, but almost always with reluctance. Why then should there be laws against their doing what they seldom wish to do, and that their own nature cries out against?

But the gross injustice and wrong in the law is yet to be shown. Every day brings instances of it. Here is a young girl, an orphan with two or three younger brothers and sisters to look after. She is ambitious for them and herself. She might earn perhaps $6 a week as a seamtress or in a store; if competent, $30 or $40 a month in teaching, but with her skirts to drag her down, as certainly as if she had fallen into the sea with the traditional millstone about her neck, the amount that she could earn for herself and those others would be limited at the best to a very small sum. She must see the family that she yearns to keep together separated, must make a market of her beauty if she has it, which at the best will only put off the evil day but a little time, or else she must put on the

---

1. Women know this well and, therefore, cling to their drapery and loose robes, which conceal defects and reveal by defining if not displaying their figures.

2. There might be numerous examples introduced of women putting on men's clothes to accomplish an object impossible for them when handicapped with their skirts. One comes to my mind of a local nature. Some twenty years ago or more, Miss Kemble, one of the first readers or recitationists ever in the country, was in Elmira, and a guest at Haight's Hotel, then kept by Silas Haight, of pleasant memory. She was a woman of very fine presence and spirits, and like all English women enjoyed horseback riding with uncommon zest. On the afternoon of her stay, she sent down to Sam Jones, whose livery stable was about where "Shorty" Fountain's is now, for a good riding horse. One of the best was sent to her, a large black animal, spirited, but kindly and well trained to the saddle. With her attendant, Miss Kemble came down in all the glory of a silk hat and long black skirt of some heavy material, the waist fitting her superb figure as though, like Madame Vertris's shoes, it had been sewed on. She mounted, and horse and rider formed a picture that made such an impression on my youthful intellect that it will never be obliterated. It was like an engraving in a fashion magazine. The party, for Miss Kemble was accompanied by two or three gentlemen started off in fine style, the admiration of quite a throng, for a lady on horseback was not so frequent an object as it is now in the streets of Elmira. They went up Lake street in good order, but arriving just a little above what is now the Lake Street Presbyterian Church, Miss Kemble's horse stopped short and refused to proceed. She urged him on with whip and spur, but all to no purpose. The animal manifested a desire to turn around, or if not allowed to do that, seemed quite disposed to lie down. One of the gentlemen alighted and examined thoroughly the trappings of the horse, but could finding nothing out of the way. Miss Kemble showed an amused vexation for some minutes and then turning the horse about galloped back with her escort to the hotel. There was a slight frown on her brow and her lips set closer together than usual, as after giving some directions to the gentleman who held the bridle of her horse, she gathered her heavy skirts in her hand and went up stairs to her room. At about the same time that the gentleman with the horse saddled for a man to ride trotted up to the Market street entrance to the hotel, Miss Kemble came down stairs again. She had no heavy skirts on to impede her movements, but was dressed in a garment of stuff so dark as to be almost black, made something like a gentleman's frock coat. The waist was not materially different from that of the dress she had previously worn, except that it had two rows of bright silver buttons running down the breast, similar ones of the cuffs and two at the waist on the back. The skirts open in front and behind, from the waist downward, were a little fuller than those of a gentleman's coat, and reached almost to the knee. She wore boots, the legs of which fitted loosely and reached above the knee. She had not changed her hat. As with but slight assistance from her attendant, she sprang astride her horse, I remember getting just a glimpse of a dark maroon pair of breeches that were fitted snugly to the leg. In a minute more they were off up Lake street, and her horse obeyed her this time, although he stopped as he did before at about the same place and seemed determined to return or lie down.

clothes of a man and earn a man's wages. Does it argue her impurity that she chooses this latter? Very far from that. It shows to the contrary. She forthwith "gets a good job." Her men's clothes do that much for her. Her sprightliness, willingness, faithfulness and patience do all the rest. She advances with her quickness of apprehension and the little ones for whom she cares are well provided for. But one day, alas! in some way or other, the discovery is made that this trustworthy, faithful person is not what she seems, so far as her sex is concerned. It is said that she has unsexed herself. Her tried ability goes for nothing. She is arrested and locked up. Why? It makes one's blood tingle to think of it. Locked up as if she had been guilty of a crime. In what, pray, has she offended? Whom has she wronged? Then she is brought before a magistrate, who reprimands her, admonishes her to assume her proper (?) attire and she is set free, with a stain upon her, for which she is not to blame, to go over the question again as to how she will keep the little family together. If she braves the matter, resumes the clothes that she was forbidden to wear, and is again arrested, she is probably sent to some Reformatory or Home, as incorrigible. Then the public are obliged to provide for both her and her little family, adding a burden that she was only too anxious to relieve them of, and was doing, as long as she was let alone. This is not an imaginary case, and similar ones are occurring every day, frequently forcing, positively forcing girls into ways of life that they were trying their best to escape.

Instances can be multiplied, of widows supporting their families and doing a man's work in a man's clothes for a man's wages, until their sex by some untoward accident, was discovered, and their support cut away from under them, but it would be unnecessary to adduce them to show to the most inconsiderate the wrong that exists. This, with the one I have already discussed in a former number of the BISTOURY, are glaring outrages on common sense, that need to be remedied, and it is to be hoped that they speedily will be.

One great principle that really underlies the structure of our Republic, and which may be expressed strongly, even if in homely language, "Give every one a chance," if applied to questions similar to these which I have barely pointed out, would obviate every difficulty. Let every one say what he chooses and do as he chooses so long as he does not interfere with his neighbor, and let it be applied to women as well as to men, and we would reach nearer to happiness than by any other course.

